# 4430 Coffee Shops and Fast-food Restaurants: Potential Neighborhood Resources for Cognitive Health and Wellbeing Among Aging Americans

**DOI:** 10.1017/cts.2020.57

**Published:** 2020-07-29

**Authors:** Jessica Finlay, Michael Esposito, Sandra Tang, Iris Gomez-Lopez, Dominique Sylvers, Suzanne Judd, Philippa Clarke

**Affiliations:** 1 University of Michigan School of Medicine; 2 University of Michigan; 3 University of Alabama at Birmingham

## Abstract

OBJECTIVES/GOALS: Environmental factors may significantly increase the risk of or buffer against Alzheimer’s disease and related dementias, yet strategies to address cognitive decline and impairment to date largely overlook the role of neighborhoods. This mixed-methods study is the first to examine potential links between access to eateries and cognitive function. The goal is to inform place-specific interventions to help aging individuals reduce risk for cognitive impairment through neighborhood community and design. METHODS/STUDY POPULATION: Following an exploratory sequential mixed-methods design, seated and mobile interviews with 125 adults aged 55-92 (mean age 71) living in the Minneapolis (Minnesota) metropolitan area suggest that eateries, including coffee shops and fast-food restaurants, represent popular neighborhood destinations for older adults and sources of wellbeing. To test the hypothesis that these sites, and the benefits they confer, are associated with cognitive welfare, we analyzed data from urban and suburban dwelling participants in the REasons for Geographic And Racial Differences in Stroke (REGARDS) study, a national racially diverse sample of older Americans followed since 2003 (n = 16,404, average age at assessment 72 years). RESULTS/ANTICIPATED RESULTS: Qualitative thematic analysis of how older adults perceived and utilized local eateries include sites of familiarity and comfort; physical and economic accessibility; sociability with friends, family, staff, and customers; and entertainment (e.g., destinations for outings and walks, free newspapers to read). Quantitative results from multilevel linear regression models demonstrate a positive association between density of eateries and cognitive functioning. Taken together, these results complicate our understanding of fast-food settings as possible sites of wellbeing through social interaction and leisure activities. DISCUSSION/SIGNIFICANCE OF IMPACT: The results contribute new evidence towards an emerging ecological model of cognitive health. Understanding whether and how retail food environments can help buffer against cognitive decline among older adults provides novel opportunities to promote wellbeing in later life through community interventions and neighborhood design.

